# Manufacturing Elements with Small Cross-Sections of 17-4 PH Steel (1.4542) with the Application of the DMLS Additive Manufacturing Method

**DOI:** 10.3390/ma14123256

**Published:** 2021-06-12

**Authors:** Grzegorz Budzik, Łukasz Przeszłowski, Tomasz Dziubek, Małgorzata Gontarz, Mariusz Dębski, Emil Smyk

**Affiliations:** 1 Department of Mechanical Engineering, Rzeszow University of Technology, al. Powstańców Warszawy 12, 35-959 Rzeszow, Poland; gbudzik@prz.edu.pl (G.B.); tdziubek@prz.edu.pl (T.D.); m.gontarz@prz.edu.pl (M.G.); m.debski@prz.edu.pl (M.D.); 2 Faculty of Mechanical Engineering, UTP University of Science and Technology in Bydgoszcz, al. prof. S.Kaliskiego, 85-796 Bydgoszcz, Poland; emil.smyk@utp.edu.pl

**Keywords:** additive manufacturing, DMLS, radiator, quality control, elements with small cross-sections

## Abstract

The application of direct metal laser sintering renders it possible to manufacture models with complex geometries. However, there are certain limits to the application of this method connected with manufacturing thin-walled cuboidal elements, as well as cylinders and holes with small diameters. The principal objective of the research was to determine the accuracy of manufacturing geometries with small cross-sections and the possibility of application in heat exchangers that are radiators with radially arranged ribs. To that end, four specimens were designed and manufactured; their geometries of representations assumed for the purpose of research (analysis) changed dimensions within the following scope: 10–0.1 mm. The specimens to be applied in the research were manufactured with 17-4 PH stainless steel (1.4542) with the application of 3D-DMLS printing and an EOS M270 printer. The measurement of accuracy was performed with the application of an optical stereomicroscope (KERN OZL-466). In addition to that, research into the chemical composition of the material, as well as the size of spherical agglomerates, was conducted with the application of a scanning electron microscope. The analysis of the chemical composition was conducted as well (after the sintering process). The analysis of the results based on the values received by means of measurements of the manufactured geometries was divided into three parts. Based on this, it is possible to conclude that the representation of models manufactured with the application of DMLS was comparable with the assumptions, and that the deviations between a nominal dimension and that received in the course of the research were within the following scope: 0–0.1 mm. At the final stage of research and based on the received results, two heat exchangers were manufactured.

## 1. Introduction

Throughout recent years, it has been possible to observe a significant development in rapid prototyping methods, which is contributed to by an ever-greater interest of numerous industries such as the aviation industry [1,2,3,4,5,6,7,8,9,10]. In practice, DMLS (direct metal laser sintering) is an alternative to models manufactured with the application of casting processes. It consists of sandwich direct metal laser sintering with the application of a fiber ytterbium laser [11,12]. The material is taken from a dispenser and afterward placed in layers with the application of a drift fender in the working space (height of a layer is dependent upon the kind of material in a dispenser; possible range: 10–80 µm). Parts are manufactured directly based on a three-dimensional computer aided design (3D CAD) model (after appropriate data processing—recording in the “stl” format), owing to which it is possible to shorten the time required to manufacture single items or short lots. An additional advantage is that it renders it possible to avoid so-called indirect modeling, and, consequently, to reduce the inaccuracy of dimensions. This method renders it possible to manufacture models with a complex geometry, frequently impossible to manufacture with the application of conventional manufacturing methods [13,14]. Unfortunately, however, limits to the application of this method connected with manufacturing thin-walled cuboidal elements, and also cylinders and holes with a small diameter, ought to be taken under consideration. It is also a very important aspect to manufacture such geometries with appropriate accuracy, and also to ensure allowances for further post-processing [15,16].

**Figure 1:**
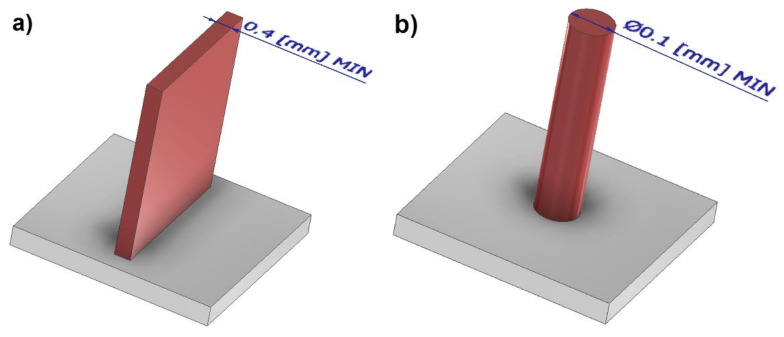
Elements with small cross-sections: (**a**) cuboidal; (**b**) cylindrical.

The objective of this article was to present the possibilities of manufacturing geometries with small cross-sections of 17-4 PH stainless steel (1.4542) with the application of 3D-DMLS printing and an EOS M270 printer. In the literature of the subject, it is possible to find information relevant to designing selected geometries for the needs of additive manufacturing with the application of a selected method of it. In the case of cuboidal structures, it is most frequently the minimum thickness of an unsupported wall that is referred to; it amounts to 0.4 mm (Figure 1a). The situation is similar in the case of unsupported cylindrical structures, yet, as far as they are concerned, the diameter amounts to 0.1 mm (Figure 1b) [17,18,19,20,21].

**Figure 2:**
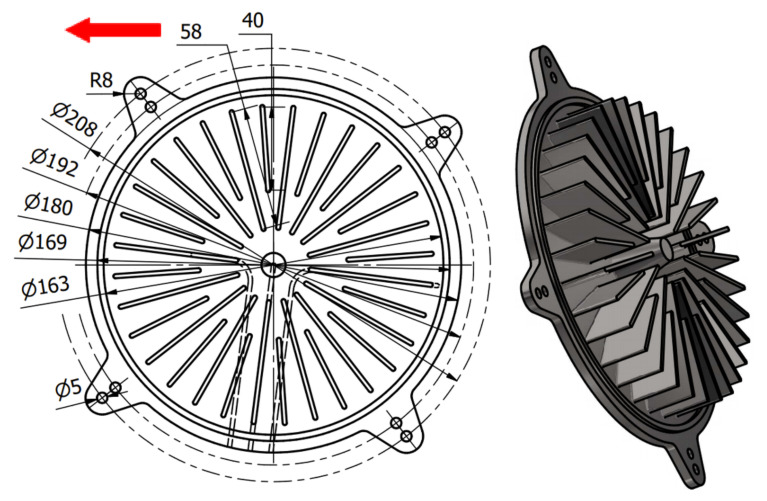
Illustrative drawing of radiator variant No. 1.

**Figure 3:**
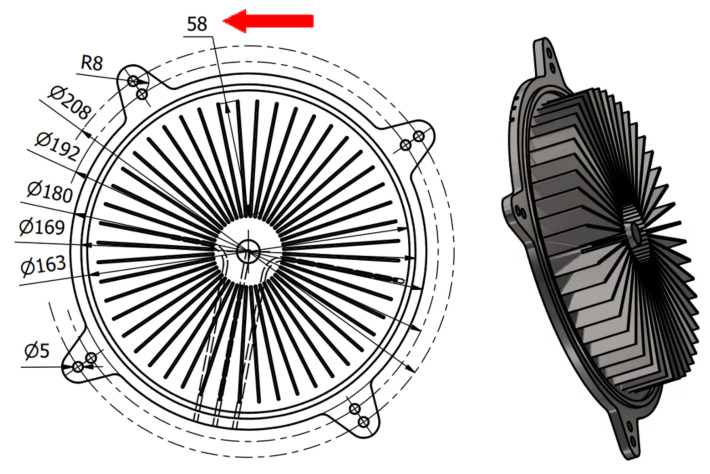
Illustrative drawing of radiator variant No. 2.

The research was conducted to manufacture heat exchangers radiators with radially arranged ribs with the application of 3D printing. The radiators were designed in two variants presented in Figure 2 and Figure 3. The principal assumptions in the course of designing a radiator were a heat exchange surface exceeding 0.1 m^2^, the number of ribs, their radial arrangement, and limits connected with the size of the working space of an EOS M270 printer [22].

In addition to that, in Figure 2 and Figure 3, the location of the XY plane, as well as the direction of a drift fender movement (red arrow) in the working space of an additive device, is presented.

## 2. Materials and Methods

### 2.1. Materials

**Figure 4:**
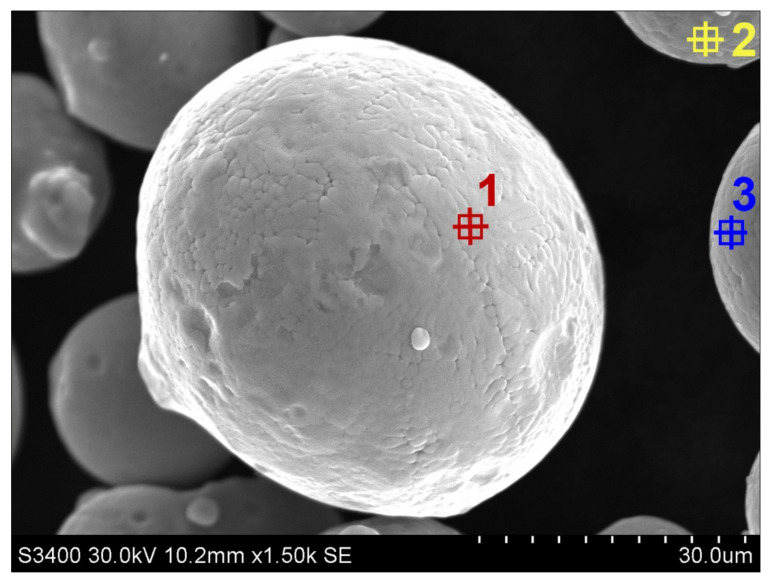
Example of 17-4 PH stainless-steel spherical particles (1,2,3- measurement points).

**Figure 5:**
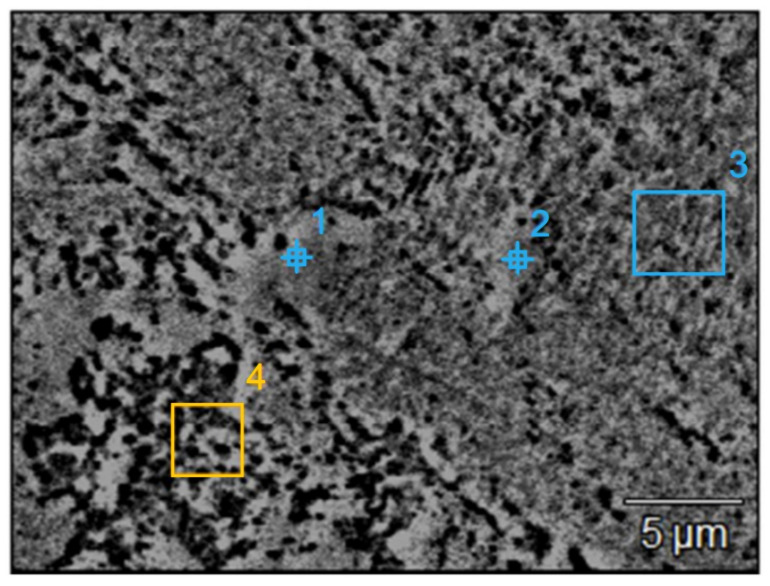
The area of microanalysis of the chemical composition of SEM sample after sintering (1,2- measurement points; 3,4- measurement areas).

For manufacturing specimens with the application of DMLS, the 17-4 PH stainless steel (1.4542) of the EOS company (trade name: GP1) (EOS GMBH, Krailling, Germany) was applied as a powder in the form of spherical particles with a grain size between 20 and 80 µm. To verify the data provided by the producer and relevant to the chemical composition of a material (Table 1), as well as to the size of the spherical particles, a scanning electron microscope was applied to research the powder (Figure 4). In addition to that, the analysis of the chemical composition after the sintering process was conducted (Table 2). The area of the microanalysis of chemical composition SEM is presented in Figure 5. In Table 3, the selected mechanical properties of the 17-4 PH stainless steel, applied in the course of manufacturing the analyzed research models, are collated. The presented data are available on the website of the producer.

### 2.2. DMLS Part Manufacturing

Specimens to be applied in the research were manufactured of the powder of the high-chromium DMLS steel with the application of an EOSINT M270 printer (EOS GMBH, Krailling, Germany) and designed in the Autodesk Inventor Professional 2020 environment (Autodesk, Inc., Mill Valley, CA, USA), to be exported afterward to an STL surface format. The base size and the location of particular elements of the researched geometry were selected to render it possible to manufacture the details in question with the application of direct metal laser sintering [16,17,23]. For the purpose of analyzing the accuracy of the representation of the geometry of prototypes manufactured with the application of DMLS, four research models (marked as presented in Table 4) were applied. The rationale behind the designed geometry of the specimen is the time required for printing. Manufacturing a single heat exchanger requires approximately 80 h, which, in the case of the conducted research, would result in a significantly extended manufacturing time and in an increased consumption of the material.

**Figure 6:**
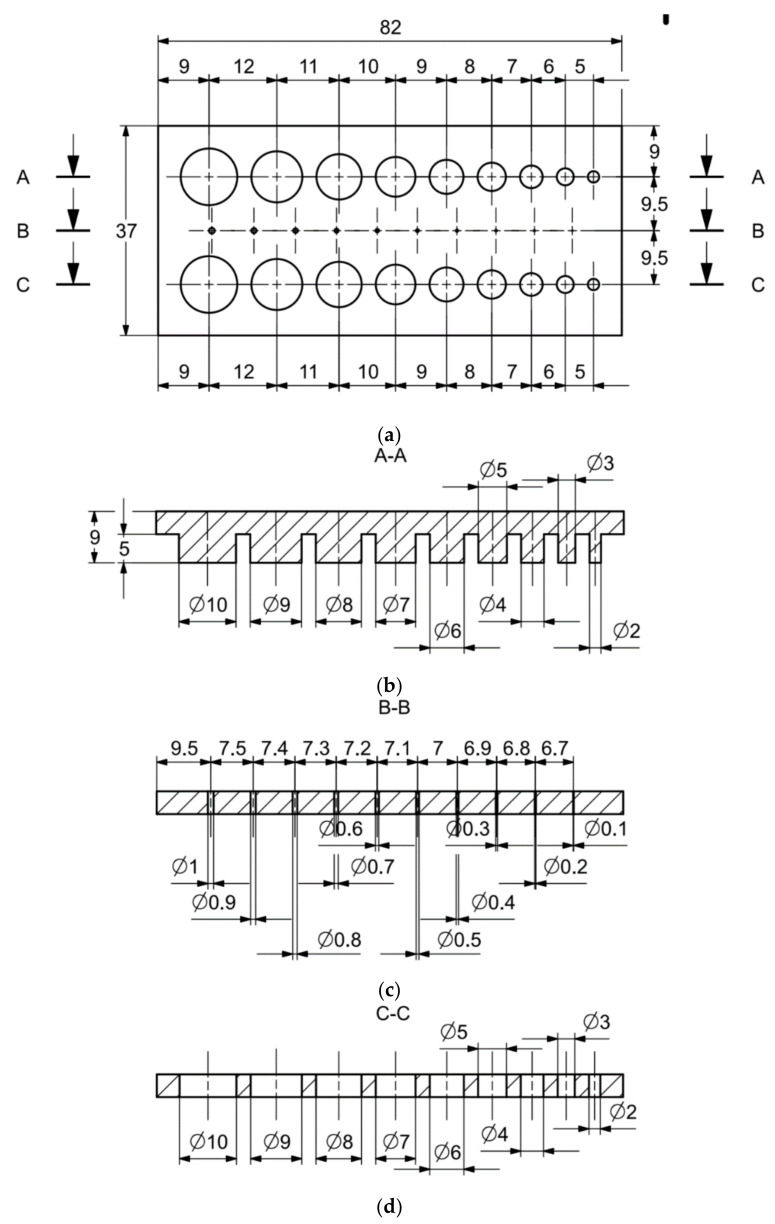
(**a**) The main view of the illustrative drawing of samples covered by the research is marked as MB1. (**b**) The A-A cross-section of cylinders Ø10–Ø2 mm based on Figure 6a of the main view of an illustrative drawing of samples covered by the research is marked as MB1. (**c**) The B-B cross-section of holes Ø1–Ø0.1 mm based on Figure 6a of the main view of an illustrative drawing of samples covered by the research is marked as MB1. (**d**) The C-C cross-section of holes Ø10–Ø2 mm based on Figure 6a of the main view illustrative drawing of samples covered by the research is marked as MB1.

**Figure 7:**
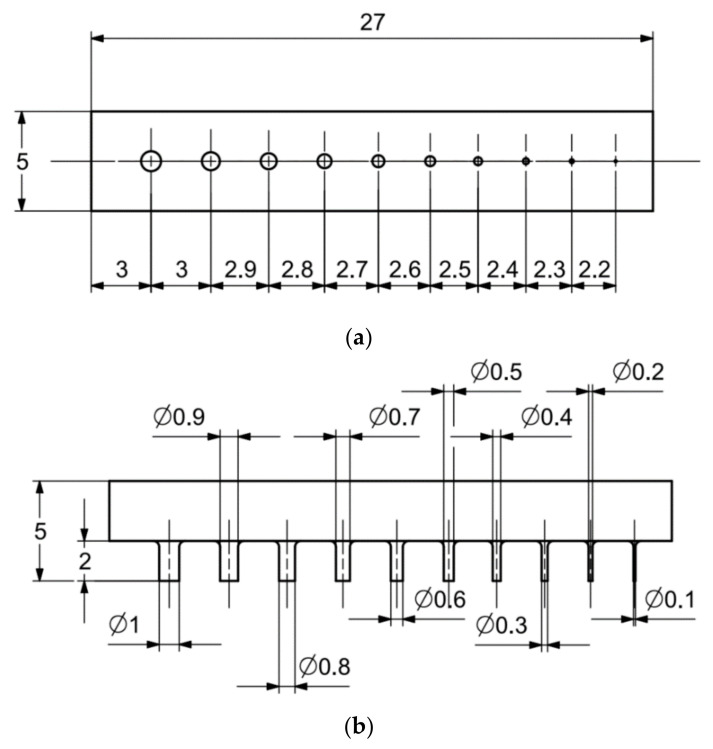
(**a**) The illustrative drawing of samples covered by the research is marked as MB2. (**b**) The top view of cylinders Ø1–Ø0.1 mm based on Figure 7a of the main view of an illustrative drawing of samples covered by the research is marked as MB2.

In Figure 6a–d, the dimensions of the specimen marked MB1 are presented. In the upper part of the specimen, there are cylinders with the following diameters: Ø10–Ø2 mm, with a step of 1 mm (from the left—cross-section A-A—Figure 6b), and below, there are holes with the following diameters: Ø1–Ø0.1 mm, with a step of 0.1 mm (from the left—cross-section B-B—Figure 6c). In turn, in the lower part of the object presented in cross-section C-C (Figure 6d), there are holes with the following diameters: Ø10–Ø2 mm, with a step of Ø1 mm. In Figure 7a,b, cylinders with the following diameters: Ø1–Ø0.1 mm, with a step of 0.1 mm, with the specimen marked MB2 from the left, are presented.

**Figure 8:**
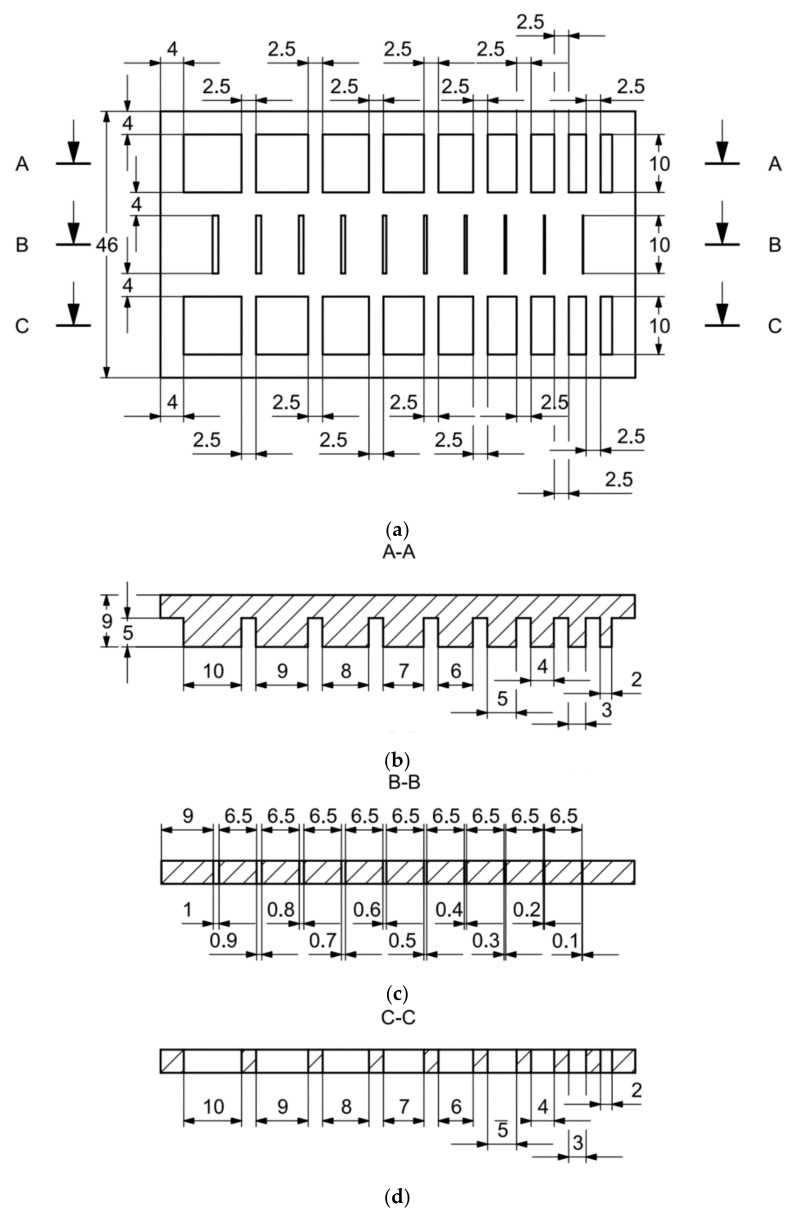
(**a**) The main view of the illustrative drawing of samples covered by the research is marked as MB3. (**b**) The A-A cross-section of solids 10–2 mm based on Figure 8a of the main view of an illustrative drawing of samples covered by the research is marked as MB3. (**c**) The B-B cross-section of cuboidal holes 1–0.1 mm based on Figure 8a of the main view of an illustrative drawing of samples covered by the research is marked as MB3. (**d**) The C-C cross-section of cuboidal holes 10–2 mm based on Figure 8a of the main view of an illustrative drawing of samples covered by the research is marked as MB3.

**Figure 9:**
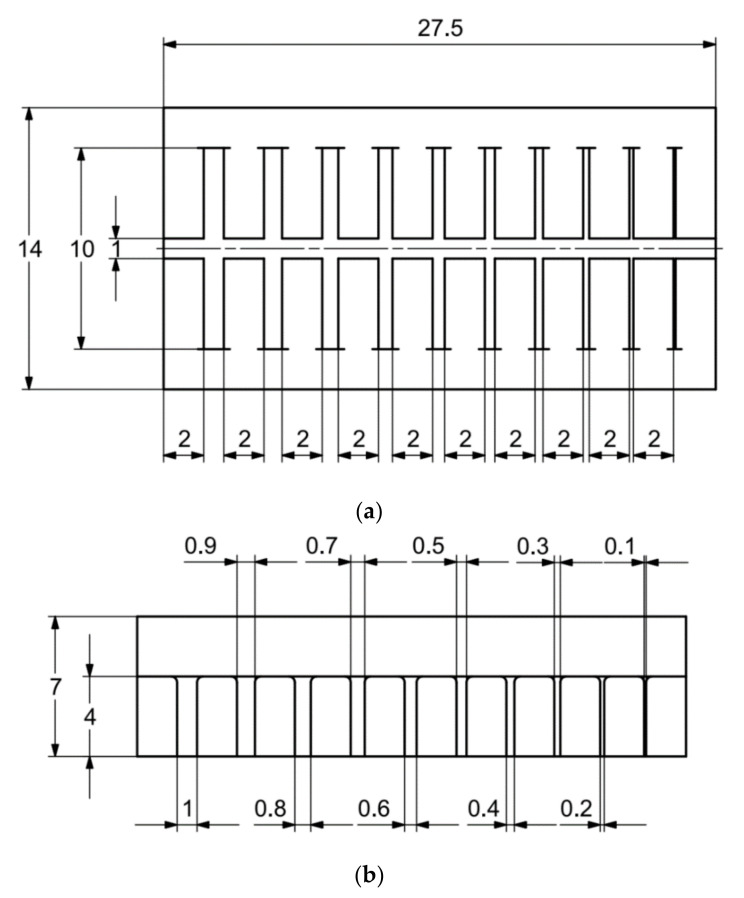
(**a**) The main view of the illustrative drawing of samples covered by the research is marked as MB4. (**b**) The top view of cuboidal objects 1–0.1 mm based on Figure 9a of the main view of an illustrative drawing of samples covered by the research is marked as MB4.

In the case of the research models marked MB3, as well as MB4, presented in Figure 8 and Figure 9, it is solely the width of the geometry that is changed. In the upper part of the specimen marked MB3 (Figure 8a), from the left, there are cuboidal objects with the following widths: 10–2 mm (with a step of 1 mm—cross-section A-A—Figure 8b), and below them, there are holes with the following widths: 1–0.1 mm, with a step of 0.1 mm (cross-section B-B—Figure 8c), and, in the lowest part of the discussed element, there are holes with the following dimensions: 10–2 mm with a step of 1 mm (cross-section C-C—Figure 8d). In turn, in Figure 9a,b, there are cuboidal objects with the following widths (from the left): 1–0.1 mm (with a step of 0.1 mm).

The models presented in Figure 6, Figure 7, Figure 8 and Figure 9 were manufactured of the powder of the high-chromium GP1 steel (EN 1.4572, 17-4 PH) with the application of an EOS M270 printer. Prior to starting the printer, “stl” model files were read in the Magics program of the Materialise company. In this program, the best possible location of the details in the virtual working space of a prototyping device was determined, and the supporting structures were defined. After verifying the correctness of the model representations, the program was applied to generate an “sli” file, containing, among others, information on the layer height (20 µm) and the location of the models on the working plane of the prototyping device, and also a “cli” file, with information on the supporting structure. Afterward, the file with information on the support was exported to an “sli” file.

In the course of pre-processing, a printing device was prepared: a laser was heated up, material residues were removed, steel powder was prepared for printing, and the work table was leveled. Afterward, the “sli” files were transferred to a program dedicated to the manufacturing process on an EOS M270 printer. This program renders it possible to determine such parameters of sintering as laser rapidity in the course of contour sintering, and also fillings, the height of the sintered layer, or laser power in the course of scanning the appropriate areas. The basic parameters of sintering are presented in Table 5.

At the further stage (post-processing), the received research models underwent initial purification and were also removed from the printer work table with the application of a hack-saw. Only the model-supporting structures were subjected to grinding.

### 2.3. Research Methodology Relevant to Geometrical Parameters

The measurements of elements were performed with the application of an optical stereomicroscope (KERN OZL-466, KERN & SOHN GMBH, Balingen, Germany), which was calibrated for every researched height. Every considered size was measured three times. Based on the received measurement results, the mean value of the researched size was calculated [24,25,26,27,28,29,30,31,32,33,34,35,36,37,38,39].

**Figure 10:**
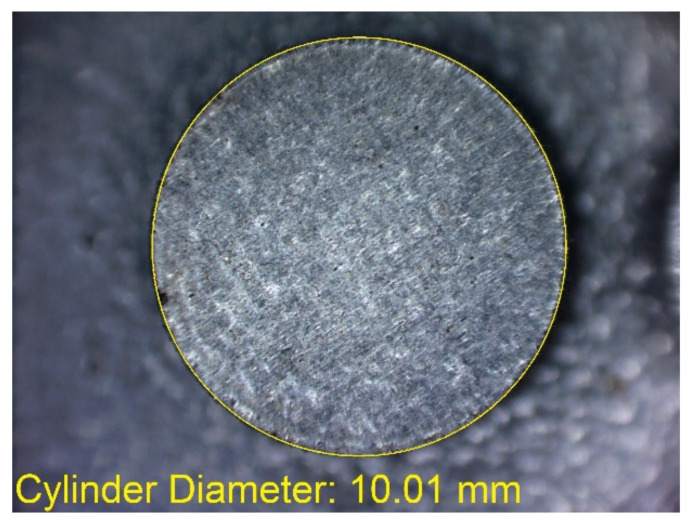
Measurement result for cylindrical holes and solids made in the DMLS technology: cylinder with a nominal diameter D_d_ = Ø10 mm (D_d1_ = 10.01 mm).

**Figure 11:**
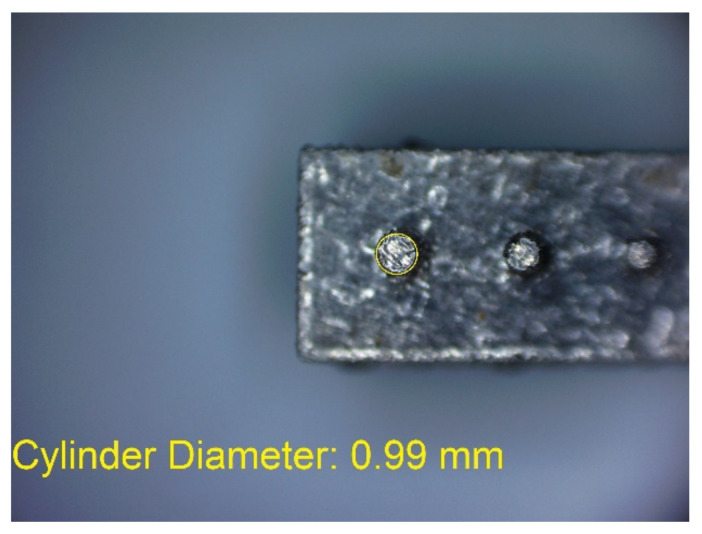
Measurement result for cylindrical holes and solids made in the DMLS technology: cylinder with a nominal diameter D_m_ = Ø1 mm (D_m1_ = 0.99 mm).

**Figure 12:**
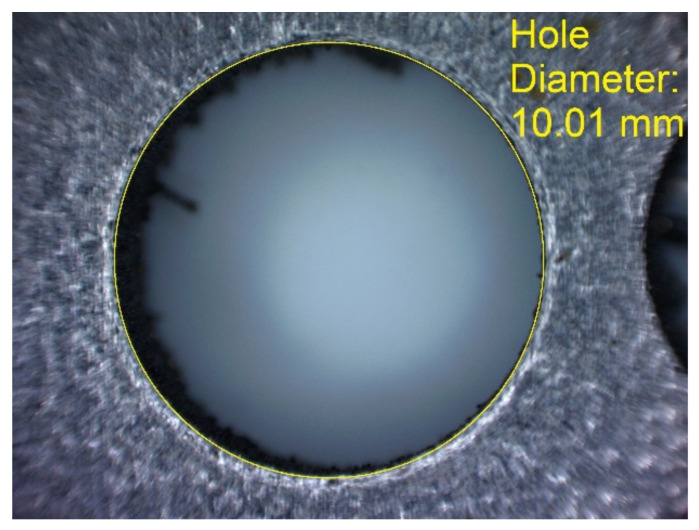
Measurement result for cylindrical holes and solids made in the DMLS technology: hole with a nominal diameter D_d_ = Ø10 mm (D_d1_ = 10.01 mm).

**Figure 13:**
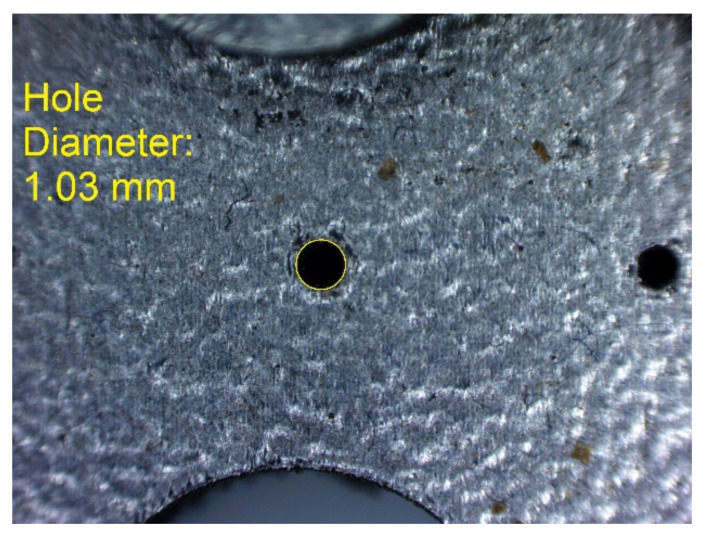
Measurement result for cylindrical holes and solids made in the DMLS technology: hole with a nominal diameter D_m_ = Ø1 mm (D_m1_ = 1.03 mm).

**Figure 14:**
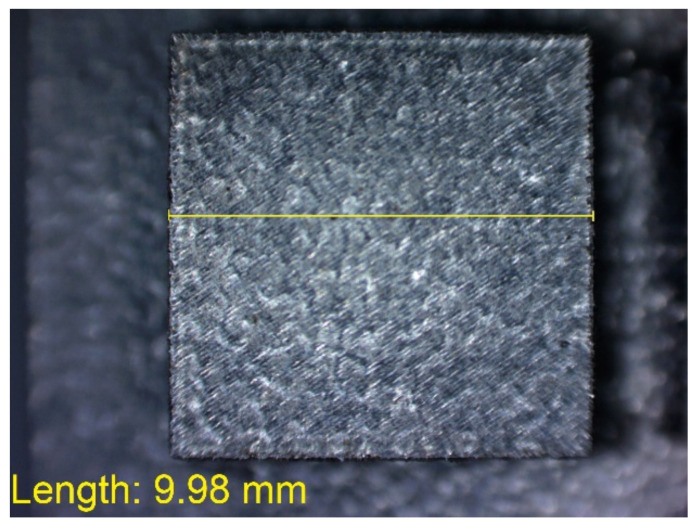
Measurement result for cuboidal holes and solids made in the DMLS technology: solid with a nominal width of 10 mm (L_dl_ = 9.98 mm).

**Figure 15:**
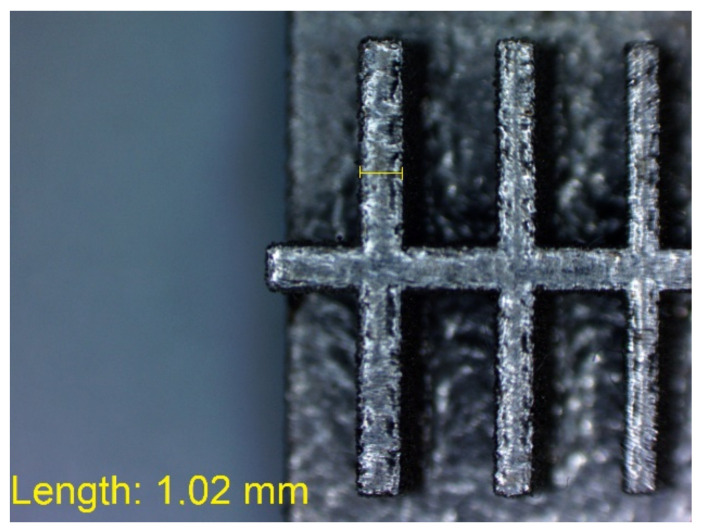
Measurement result for cuboidal holes and solids made in the DMLS technology: solid with a nominal width of 1 mm (L_m1_ = 1.02 mm).

**Figure 16:**
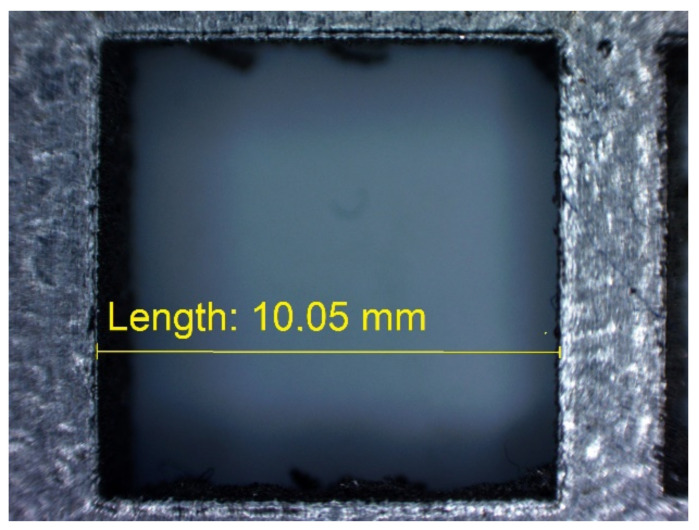
Measurement result for cuboidal holes and solids made in the DMLS technology: cuboidal hole with a nominal width of 10 mm (L_dl_ = 10.05 mm).

**Figure 17:**
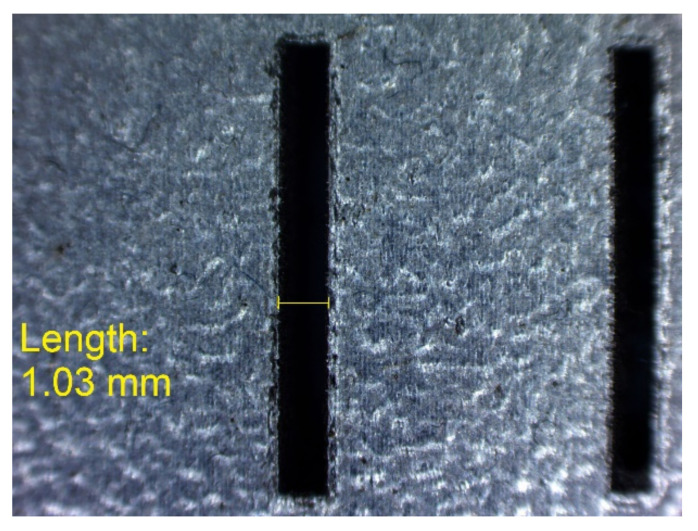
Measurement result for cuboidal holes and solids made in the DMLS technology: cuboidal hole with a nominal width of 1 mm (L_m1_ = 1.03 mm).

In Figure 10, Figure 11, Figure 12, Figure 13, Figure 14, Figure 15, Figure 16 and Figure 17, the method of the measurement of cylindrical elements, and of the rectangular ones, manufactured with the application of DMLS, on which it is possible to observe the model structure (such as detail surface and the quality of workmanship), is presented. The holes, as well as the rectangular objects (Figure 14, Figure 15, Figure 16 and Figure 17), were measured three times in the center and at both ends of the measured element.

## 3. Results and Discussion

In Table 6, the received results relevant to manufactured prototypes measured with the application of an optical microscope, together with the calculated values of arithmetic means of the measured parameters, as well as deviations between the nominal dimension and the received mean values, are collated.

### 3.1. Comparison of the Obtained Results

The results received in the course of the research were divided into three parts. The first included those measurements that were divided in terms of the researched parameter, and the objective of that was to demonstrate differences between the nominal dimension (that assumed in the course of designing the details) and that received in the course of research.

**Figure 18:**
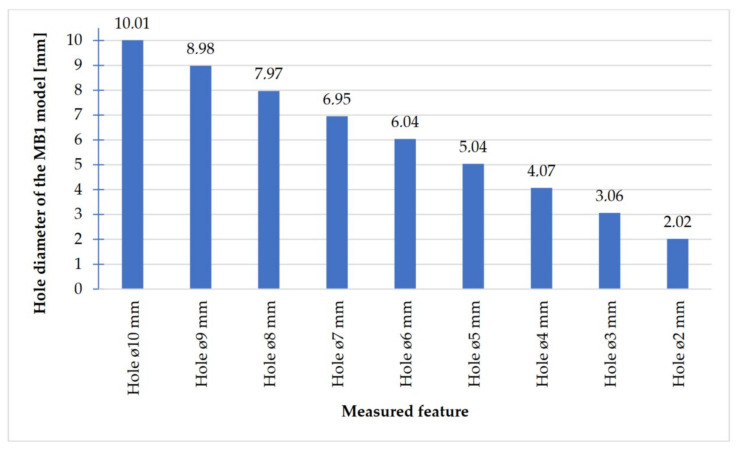
Average values calculated based on the cylindrical holes measurement results with dimensions Ø10–Ø0.2 mm.

**Figure 19:**
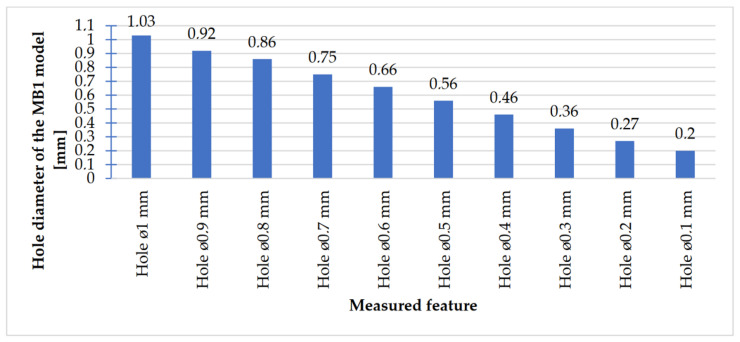
Average values calculated based on the cylindrical holes measurement results with dimensions Ø1–Ø0.1 mm.

**Figure 20:**
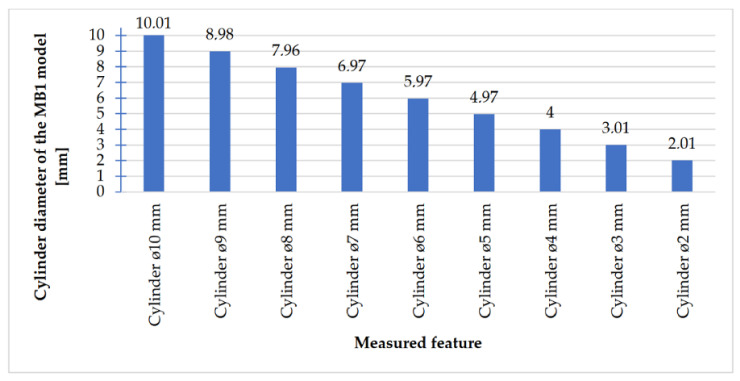
Average values calculated based on the cylinders measurement results with dimensions Ø10–Ø2 mm.

**Figure 21:**
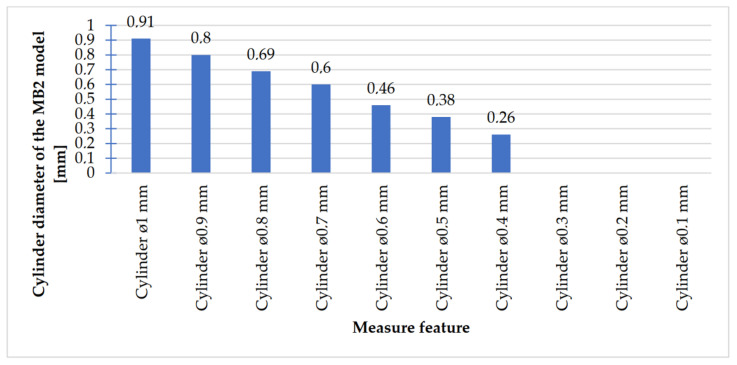
Average values calculated based on the cylinders measurement results with dimensions Ø1–Ø0.1 mm.

In Figure 18, Figure 19, Figure 20 and Figure 21, the received mean values of the result measurements relevant to cylindrical elements, and the names of the researched parameters that match the nominal dimension assumed in the course of designing research models, are presented.

**Figure 22:**
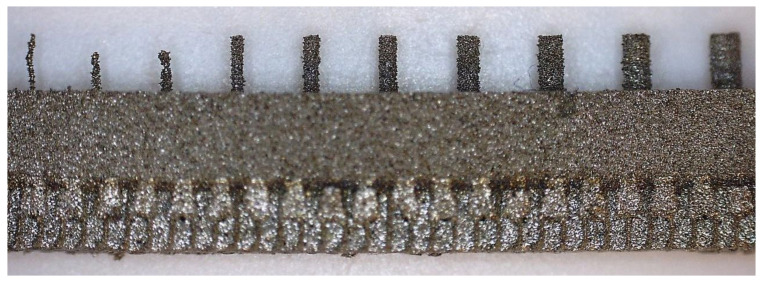
Front view of the model of cylinders with diameters of Ø1–Ø0.1 mm made of 17-4 PH steel.

Comparing the values received in the course of the measurements of cylindrical holes with the dimension assumed in the course of designing details that are presented in Figure 6c,d, it is ascertained that they were comparable with the nominal dimensions of the researched parameters. All the holes within the considered range were manufactured in research details. The situation was the same in the case of cylinders (Figure 7a,b), where the measured values were also similar to the nominal dimension, wherein the following range of cylinders: Ø1–Ø0.1 mm, the accuracy of workmanship and reducing cylinder diameter was decreasing. It is also worth adding that all the cylinders having the assumed diameters were manufactured with the application of the analyzed DMLS; however, the height of the cylinders (namely, Ø0.3, Ø0.2, and Ø0.1 mm) was lower than those of the other ones, which resulted in it being impossible to calibrate a research device to fit their height, which is presented in Figure 22.

**Figure 23:**
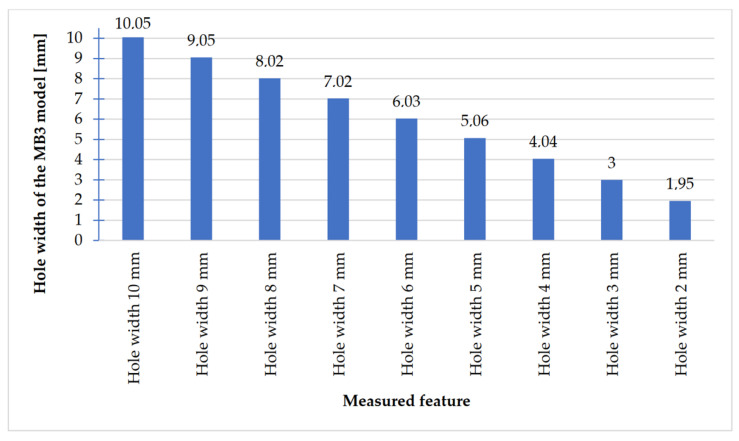
Values measured for rectangular holes 10 to 2 mm wide.

**Figure 24:**
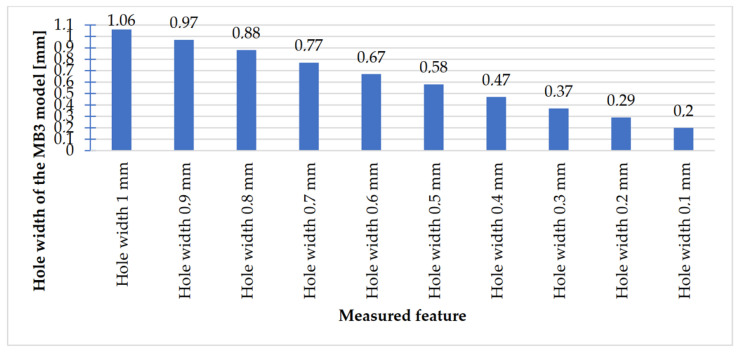
Measured values of rectangular holes in the range of 1–0.1 mm.

**Figure 25:**
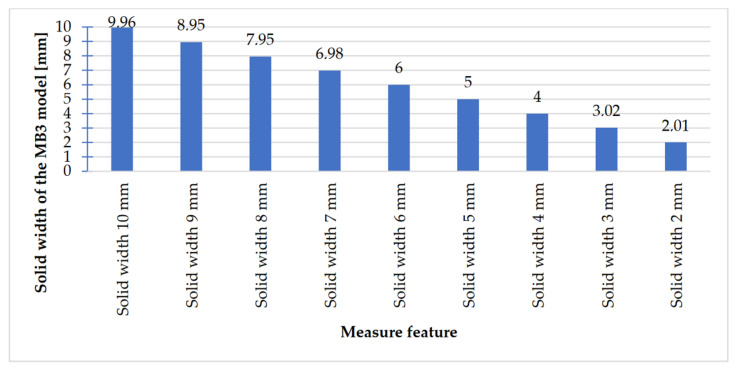
Measured values of rectangular solids with dimensions 10–2 mm.

**Figure 26:**
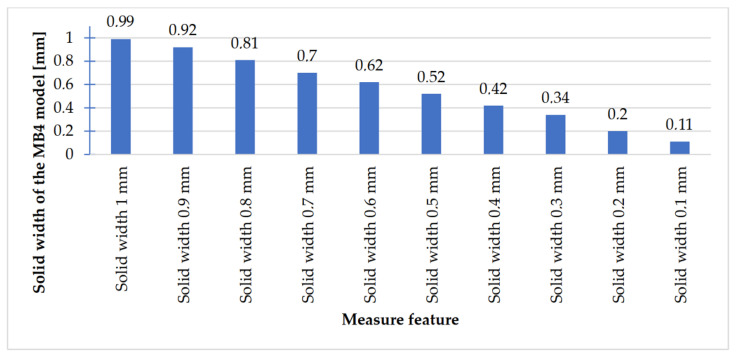
Measured values of rectangular solids with dimensions from 1 to 0.1 mm.

In Figure 23, Figure 24, Figure 25 and Figure 26, the received results of the measurements of cuboidal elements: holes, as well as objects in which the names of particular parameters match the assumed nominal dimension, are presented.

**Figure 27:**
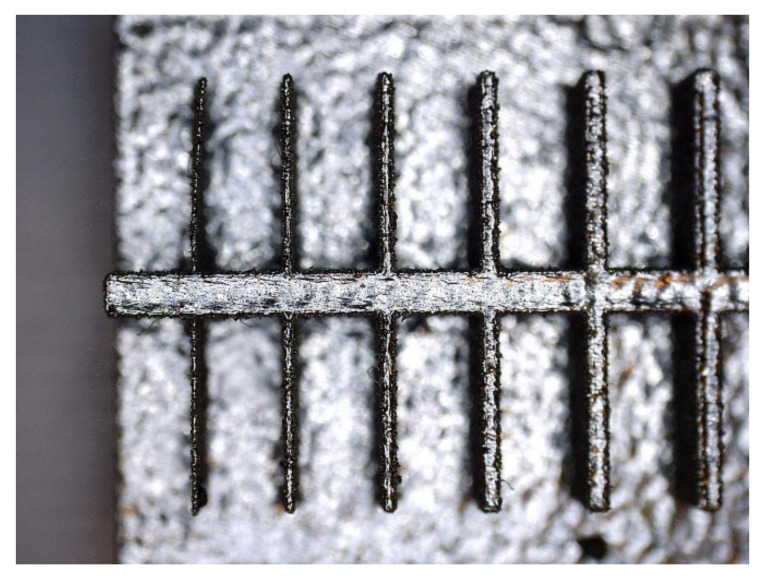
Presentation of the deformation of a fragment of a detail of a rectangular model made in the DMLS technology.

Comparing the values received in the course of the measurements of holes and rectangular objects, it is possible to observe that they are comparable with the nominal dimension for the researched parameter (Figure 8 and Figure 9). It is also possible to observe that, for the holes having the following width: 0.1–1 mm (Figure 8c), results higher than the assumed sizes of the considered parameter were received. It is also worth mentioning that a nonsignificant deformation was observed only in the case of an object having a width of 0.1 mm, which is presented in Figure 27.

### 3.2. Comparison of the Difference between the Nominal and Measured Dimension

In the second part of the elaboration of the results of research into geometrical sizes, the values of deviations between the dimension assumed and that measured for the object models, as well as cylindrical and rectangular holes, were compared.

**Figure 28:**
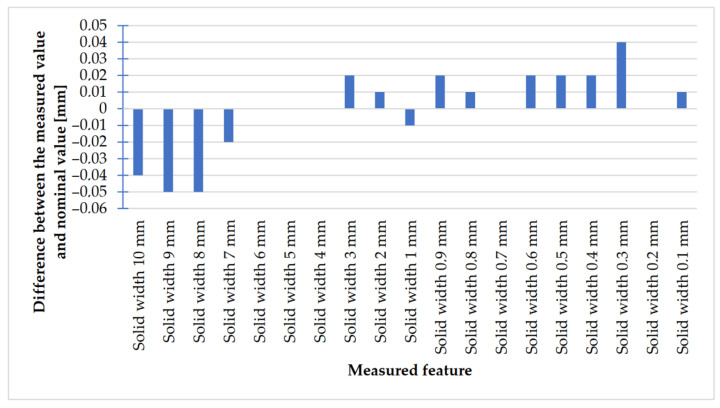
Deviation analysis for rectangular solids of variable width made in DMLS technology.

**Figure 29:**
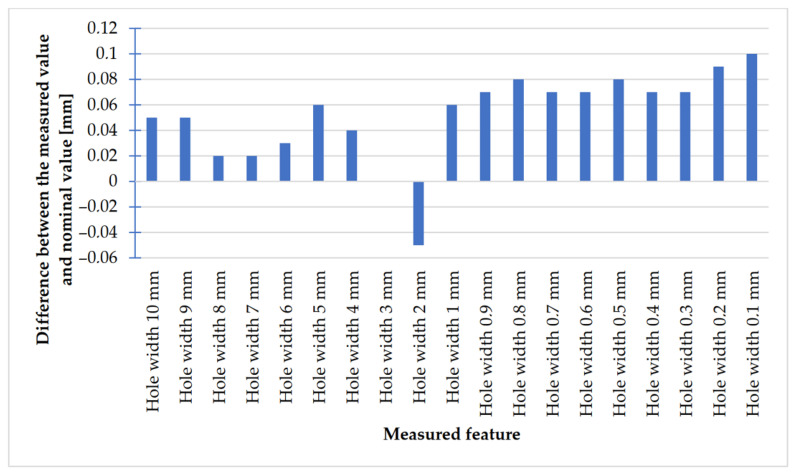
Deviation analysis for rectangular holes model made in the DMLS technology.

In Figure 28 and Figure 29, the charts of deviations for rectangular models are presented.

Looking at the chart presented in Figure 28, it is possible to observe that the values of deviations in the case of rectangular objects with the following widths: 10–7 mm were negative; in turn, the values of deviations in the case of objects with the following widths: 3–0.1 mm (except for an object with the width of 1 mm) were positive. The situation was the opposite in the case of rectangular holes (Figure 29), whose values of deviations were positive (except for holes with a width of 2 mm).

**Figure 30:**
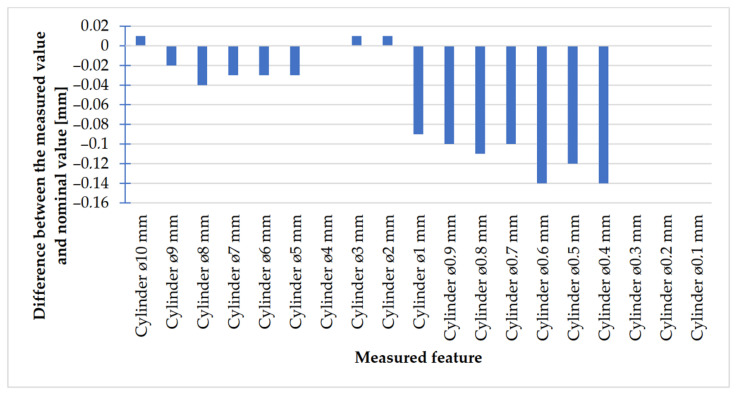
Deviation analysis for cylinders made in DMLS technology.

**Figure 31:**
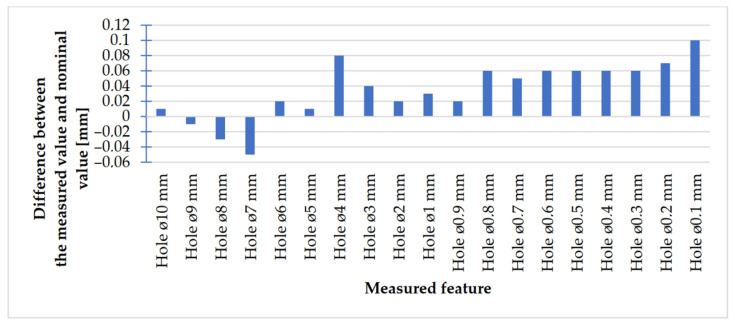
Deviation analysis for cylindrical holes with diameters from Ø10 to Ø0.1 mm of the model made in the DMLS technology.

In Figure 30 and Figure 31, the values of deviations in the case of cylindrical elements (holes and cylinders) are presented.

In the cylinder model (Figure 30), in the course of the analysis of accuracy, it is possible to observe that, in the case of a cylinder with the dimension of Ø2 mm, the values of deviations were below 0.05 mm. In the case of cylinders with diameters of Ø1 mm and smaller, the values of deviations increased, unlike in the case of cylindrical holes (Figure 31), for which holes between Ø6 and Ø0.1 mm were associated with positive values within the following scope: 0.02–0.1 mm.

### 3.3. The Analysis of the Values of Means Deviations

In the third part of the elaboration of the measurement results, attention was focused upon the comparison of the largest and smallest absolute value, as well as the mean value of differences received as a result of research into rectangular and cylindrical models, and the mean values of deviations between the nominal dimension and that measured for spherical and angular models. The described collations are presented in Table 7.

Based on the collation presented in Table 3, it is possible to observe that the greatest absolute difference between the nominal dimension and that measured in the case of cylindrical elements (both for the holes and objects) was ascertained in the case of cylinders within the following scope: Ø1–Ø0.1 mm, contrary to the smallest absolute difference, which was observed in the case of elements within the following scope: Ø10–Ø2 mm. In the case of the rectangular elements, it was impossible to observe such a correlation; however, in terms of rectangular holes, the greatest mean value of deviations between the nominal dimension and that measured for all the researched elements was observed.

**Figure 32:**
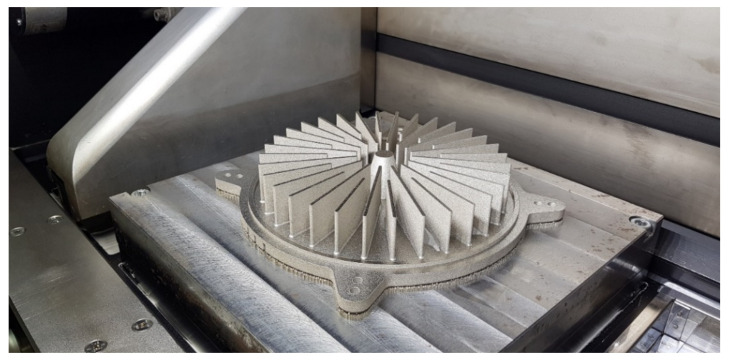
Radiator in the workspace of the EOS M270 variant No. 1.

**Figure 33:**
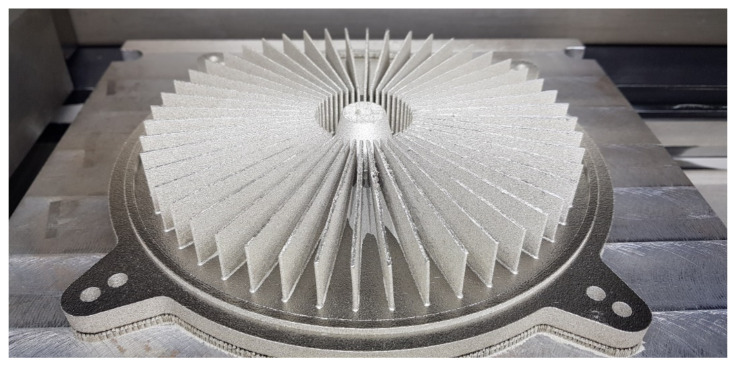
Radiator in the workspace of the EOS M270 variant No. 2.

In order to verify the received results, two radiators were referred to in the Introduction with appropriately shaped ribs with small cross-sections. In both of these cases, the ribs are arranged radially, which, in addition to anything else, may render manufacturing difficult (rake angle between a drift fender edge and a rib long edge ought not to be 0 [°]). In Figure 32 and Figure 33, the radiators manufactured with the application of the DMLS methods are presented (variants 1 and 2) and designed in accordance with the assumptions determined at the beginning of this article, and also based on received research.

## 4. Conclusions

The knowledge of the mechanical properties of materials renders it possible to design a given construction in a way that ensures that the loads caused in the course of operating it do not result in damage. It also refers to the ability of the material to be deformed in the course of being shaped. As observed based on the data contained in Table 2, the producer determined in detail and accurately the mechanical properties of the considered materials of which they had informed, which may prove that this material has been researched well. The resistance of the analyzed material is high (up to 1 GPa). The highest value for the 17-4 PH steel amounts to 40.8 HRC, which proves that the material is highly hard [40,41].

The research presented in the article was intended to show the possibility of manufacturing thin-walled structural elements that have a fulcrum only at the base. Additionally, already at the modeling stage of a complex structure, the constructor must have basic knowledge related to the specifics of the DMLS process. At this stage, the direction of the incremental forming in the DMLS method was determined, and it followed the direction of the material being applying by the recoater. Subsequently, the cross-section at the point of contact of the knife with the model should be as small as possible. It is related to the force exerted by the recoater on the model, which was minimal. Otherwise, the structure may deform or collapse, and thus, the process of forming the geometry will not process properly. Therefore, the article aimed to carry out research on the manufacture of thin-walled elements used in the construction of radiators. They were conditioned by the necessity to make radiators with an increased exchange surface through thin-walled elements such as ribs arranged in a radial manner. Manufacturing such long elements for a predetermined height in a radial manner can cause technological problems because it is impossible to arrange the research model in such a way that all the ribs are positioned in the direction of the thrust line of the recoater, which is consistent with the direction indicated by the red arrow (Figure 3).

The angle between the adjacent ribs of the radiator variant No. 2, which is presented in Figure 3, was almost zero. Thanks to the preliminary samples made with different wall thicknesses, it was possible to determine the minimum thickness of the rib and indicate the height so that the process was carried out correctly. The article presents two variants of radiator geometry (Figure 2 and Figure 3) in order to confirm the validity of the research and the assumptions made. The article also takes a practical nature, indicating tendencies in the incremental formation of elements with small cross-sections. Thanks to the obtained results, it was also possible to determine whether the obtained geometries have an “on the plus” or “on the minus” tendency in relation to the nominal dimension determined during 3D-CAD geometry modelling. The obtained data were collected and presented in the form of graphs and tables. The experimentally determined research results for thin-walled models can be a kind of guide for the constructor during modelling geometries intended for additive manufacturing. Furthermore, in this article, variants No. 1 and No. 2 of the radiator serve as examples of a functional prototype confirming the design assumptions that were determined after conducting a dimensional analysis of the MB1-MB4 research samples.

Based on the analysis of geometrical parameters contained in Table 4, it is possible to ascertain that the accuracy of the representation of prototype models is not much different from the 3D CAD model. The 17-4 PH steel is a material having high levels of mechanical properties, which is directly reflected in high-dimensional-shape accuracy of the manufactured details, in which, in the case of cylindrical and rectangular elements, deviations between the nominal dimension and that received in the course of the research were within the following scope: 0–0.1 mm.

The accuracy of manufacturing cuboidal and cylindrical research models was consistent with the parameters specified by the material manufacturer. The manufacturing of models in the range from Ø0.1 to Ø0.3 mm was problematic primarily for cylindrical models, as presented in Figure 22. However, for cuboidal models, the only deformations occurred in the model with a width of 0.1 mm, as presented in Figure 27. In the case of manufacturing holes in the research cylindrical models, their accuracy was much higher than in the case of cylindrical solids, which can be seen in Figure 18, Figure 19, Figure 20 and Figure 21, Figure 30, and Figure 31. The value of the deviation between the nominal dimension and the average value obtained during measuring elements for rectangular holes in the width range from 10 to 0.1 mm was comparable and ranged from 0.02 to 0.1 mm, with two exceptions, which have been presented in Figure 29. Taking into consideration both cylindrical and cuboidal solids, elements in the range from 7 to 2 mm were characterized by the highest accuracy, which have been presented in Figure 28 and Figure 29. Comparing the research models, cuboidal models were characterized by higher accuracy compared to the cylindrical models.

Comparing the photographs of the models taken in the course of the research stage, it was possible to ascertain that the staircase effect in the case of models manufactured with the application of DMLS was not observed and that the model structure was uniform.

## Figures and Tables

**Table 1:** The chemical composition of 17-4 PH steel (areas are shown in Figure 4).

Area	Si-K (%)	Cr-K (%)	Mn-K (%)	Fe-K (%)	Ni-K (%)	Cu-K (%)	Nb-K (%)	Ta-L (%)
1	4.3	16.9	0.9	70.8	4.0	3.0	0.0	0.0
2	2.1	17.1	1.0	72.1	4.0	3.4	0.3	0.0
3	1.3	18.2	1.1	70.1	4.3	4.2	0.6	0.1

**Table 2:** The chemical composition of DMLS sintered parts (areas are shown in Figure 5).

Area	Si-K (%)	Cr-K (%)	Mn-K (%)	Fe-K (%)	Ni-K (%)	Cu-K (%)	Nb-K (%)	Ta-L (%)
1	1.9	16.6	1.0	69.5	3.8	3.7	0.2	0.0
2	1.4	17.5	0.9	72.3	4.2	3.6	0.1	0.0
3	1.2	17.4	1.1	72.6	4.0	3.5	0.1	0.0
4	1.4	17.3	1.1	72.4	4.0	3.6	0.2	0.1

**Table 3:** Selected material data of stainless steel 17-4 PH.

Alloy designation	17-4 (United States) 1.4542 (Europe) X5CrNiCuNb16-4 (Germany)
**Geometric Data**	
Minimum recommended layer thickness [µm]	20
Typical achievable part accuracy	a. ± 20 ÷ 50 µm (small parts) b. ± 0.2% (large parts) ^1^
Minimum wall thickness [mm]	0.3 ÷ 0.4
Surface roughness	a. R_a_: 2.5 ÷ 4.5 µm, R_y_: 15 ÷ 40 µm (after shot-peening) b. R_z_ do < 0.5 µm (detail can be very finely polished) (after polishing)
**Mechanical Properties of Parts**	
Ultimate tensile strength [MPa]	a. min. 850, typical 930 ± 50 (XY); min. 850, typical 960 ± 50 (Z) ^2^ b. typical 1100 (XY); typical 980 (Z) ^3^
Elongation at break [%]	a. min. 25, typical 31 ± 5 (XY); min. 25, typical 35 ± 5 (Z) ^2^ b. typical 29 (XY); typical 31 (Z) ^3^
Modulus of elasticity (Young’s modulus) [GPa]	a. 170 ± 30 ^2^ b. typical 180 ^3^
Upper yield strength [MPa]	a. min. 595, typical 645 ± 50 (XY); min. 580, typical 630 ± 50 (Z) ^2^ b. typical 634 (XY); typical 595 (Z) ^3^
Lower yield strength [MPa]	a. min. 530, typical 586 ± 50 (XY); min. 530, typical 570 ± 50 (Z) ^2^ b. typical 590 (XY); typical 550 (Z) ^3^
Hardness	a. as built: ok. 230 ± 20 HV1, i.e., approx. 18.0 ± 1.6 HRC ok. 250 HV1 ÷ 400 HV1, i.e., approx. 22.2 ÷ 40.8 HRC

Comment: ^1^—the accuracy can be improved by post-process stress-relieving for 1 h at 650 °C; ^2^—as manufactured; ^3^—stress relieved (1 h at 650 °C).

**Table 4:** Research models.

No.	Description	Mark	View
1.	base with cylindrical holes and cylinders with diameters from Ø10 to Ø2 mm and cylindrical holes with diameters from Ø1 to Ø0.1 mm	MB1	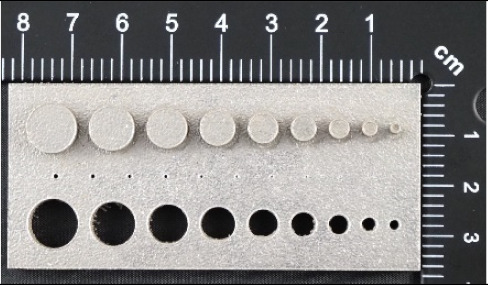
2.	base with cylinders with diameters from Ø1 to Ø0.1 mm	MB2	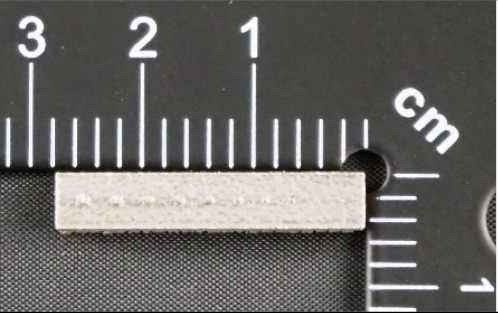
3.	base with cuboidal objects 10 to 2 mm wide and rectangular holes 10 to 2 mm wide and 1 to 0.1 mm wide	MB3	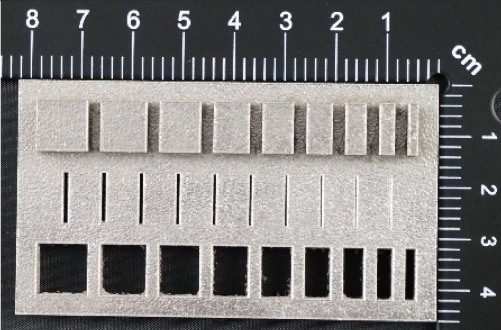
4.	base with cuboidal objects 1 to 0.1 mm wide	MB4	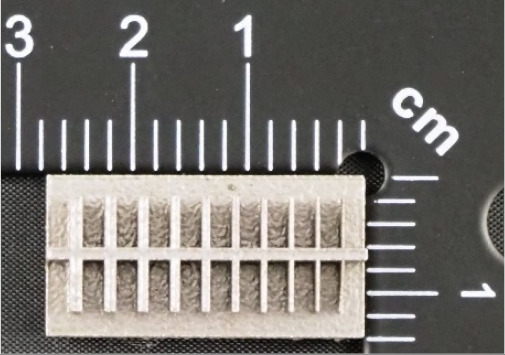

**Table 5:** Selected parameters of the sintering process in the DMLS method.

Type of Parameter	Mark	Unit	Value
Layer thickness	d	(mm)	0.2
Laser power during sintering	P_laser_	(W)	180
First contour sintering speed	v_K1_	(mm/s)	1200
Value of the first contour offset from the CAD model contour	BO_1_	(mm)	0.06
First contour sintering width	SB_1_	(mm)	0.09
Sintering speed of the initial or final layers inside the contour	v_H_	(mm/s)	1200
Width of the inner layer melt with the contour of the initial and final layers	h_H_	(mm)	0.1
Core sintering speed	v_K_	(mm/s)	1250
Width of the inner layer melt input with the core layer contour	h_K_	(mm)	0.08
Sintering speed of the second contour coinciding with the contour of the CAD model	v_K2_	(mm/s)	2200
Value of the second contour offset from the CAD model contour	BO_2_	(mm)	0.06
Second contour sintering width	SB_2_	(mm)	0.08

**Table 6:** Measurement results of the research models made in DMLS technology from 17-4 PH stainless steel.

No.	Model	Measured Feature	Actual Value (B_1_) [mm]	Measurement Results [mm]			Arithmetic Mean of the Measurement Results (B_2_) [mm]	Difference between the Arithmetic Mean and the Actual Value (B_2_-B_1_) [mm]
				1	2	3		
1	MB1	Hole Ø10 mm	10	10.01	10.01	10.01	10.01	0.01
		Hole Ø9 mm	9	8.98	9.00	8.98	≈8.99	−0.01
		Hole Ø8 mm	8	7.95	7.98	7.97	≈7.97	−0.03
		Hole Ø7 mm	7	6.95	6.95	6.95	6.95	−0.05
		Hole Ø6 mm	6	6.02	5.99	6.04	≈6.02	0.02
		Hole Ø5 mm	5	5.04	4.96	5.04	≈5.01	0.01
		Hole Ø4 mm	4	4.07	4.09	4.07	≈4.08	0.08
		Hole Ø3 mm	3	3.00	3.06	3.06	3.04	0.04
		Hole Ø2 mm	2	2.03	2.01	2.02	2.02	0.02
		Hole Ø1 mm	1	1.03	1.02	1.04	1.03	0.03
		Hole Ø0.9 mm	0.9	0.94	0.92	0.91	≈0.92	0.02
		Hole Ø0.8 mm	0.8	0.84	0.88	0.87	≈0.86	0.06
		Hole Ø0.7 mm	0.7	0.76	0.73	0.75	≈0.75	0.05
		Hole Ø0.6 mm	0.6	0.66	0.65	0.68	≈0.66	0.06
		Hole Ø0.5 mm	0.5	0.56	0.56	0.57	≈0.56	0.06
		Hole Ø0.4 mm	0.4	0.46	0.46	0.46	0.46	0.06
		Hole Ø0.3 mm	0.3	0.36	0.36	0.37	≈0.36	0.06
		Hole Ø0.2 mm	0.2	0.27	0.26	0.28	0.27	0.07
		Hole Ø0.1 mm	0.1	0.19	0.20	0.20	≈0.20	0.10
		Cylinder Ø10 mm	10	10.01	9.99	10.03	10.01	0.01
		Cylinder Ø9 mm	9	8.96	8.99	8.99	8.98	−0.02
		Cylinder Ø8 mm	8	7.96	7.97	7.96	≈7.96	−0.04
		Cylinder Ø7 mm	7	6.95	6.97	6.98	≈6.97	−0.03
		Cylinder Ø6 mm	6	5.98	5.95	5.97	≈5.97	−0.03
		Cylinder Ø5 mm	5	4.98	4.97	4.97	≈4.97	−0.03
		Cylinder Ø4 mm	4	4.00	4.01	3.99	4.00	0.00
		Cylinder Ø3 mm	3	3.00	3.00	3.02	≈3.01	0.01
		Cylinder Ø2 mm	2	2.01	2.02	2.00	2.01	0.01
2	MB2	Cylinder Ø1 mm	1	0.89	0.92	0.93	≈0.91	−0.09
		Cylinder Ø0.9 mm	0.9	0.81	0.80	0.79	0.80	−0.10
		Cylinder Ø0.8 mm	0.8	0.69	0.70	0.68	0.69	−0.11
		Cylinder Ø0.7 mm	0.7	0.61	0.59	0.60	0.60	−0.10
		Cylinder Ø0.6 mm	0.6	0.46	0.45	0.46	≈0.46	−0.14
		Cylinder Ø0.5 mm	0.5	0.38	0.35	0.40	≈0.38	−0.12
		Cylinder Ø0.4 mm	0.4	0.26	0.25	0.26	≈0.26	−0.14
		Cylinder Ø0.3 mm	0.3	cylinders impossible to measure				
		Cylinder Ø0.2 mm	0.2					
		Cylinder Ø0.1 mm	0.1					
3	MB3	Hole width 10 mm	10	10.05	10.07	10.04	≈10.05	0.05
		Hole width 9 mm	9	9.04	9.04	9.06	≈9.05	0.05
		Hole width 8 mm	8	8.03	8.01	8.03	≈8.02	0.02
		Hole width 7 mm	7	7.01	7.01	7.03	≈7.02	0.02
		Hole width 6 mm	6	6.02	6.03	6.04	6.03	0.03
		Hole width 5 mm	5	5.06	5.06	5.06	5.06	0.06
		Hole width 4 mm	4	4.05	4.00	4.06	≈4.04	0.04
		Hole width 3 mm	3	3.00	2.98	3.01	≈3.00	0.00
		Hole width 2 mm	2	1.96	1.93	1.96	1.95	−0.05
		Hole width 1 mm	1	1.03	1.06	1.10	≈1.06	0.06
		Hole width 0.9 mm	0.9	0.96	0.98	0.96	≈0.97	0.07
		Hole width 0.8 mm	0.8	0.89	0.88	0.87	0.88	0.08
		Hole width 0.7 mm	0.7	0.75	0.78	0.77	≈0.77	0.07
		Hole width 0.6 mm	0.6	0.67	0.68	0.67	≈0.67	0.07
		Hole width 0.5 mm	0.5	0.58	0.58	0.58	0.58	0.08
		Hole width 0.4 mm	0.4	0.50	0.43	0.49	≈0.47	0.07
		Hole width 0.3 mm	0.3	0.38	0.39	0.35	≈0.37	0.07
		Hole width 0.2 mm	0.2	0.30	0.30	0.28	≈0.29	0.09
		Hole width 0.1 mm	0.1	0.22	0.19	0.20	≈0.20	0.10
		Solid width 10 mm	10	9.96	9.98	9.94	9.96	−0.04
		Solid width 9 mm	9	8.95	8.94	8.95	≈8.95	−0.05
		Solid width 8 mm	8	7.95	7.93	7.97	7.95	−0.05
		Solid width 7 mm	7	6.98	6.95	7.01	6.98	−0.02
		Solid width 6 mm	6	5.99	5.99	6.02	6.00	0.00
		Solid width 5 mm	5	5.00	5.00	5.00	5.00	0.00
		Solid width 4 mm	4	4.00	4.00	3.99	≈4.00	0.00
		Solid width 3 mm	3	3.01	3.03	3.01	≈3.02	0.02
		Solid width 2 mm	2	2.00	2.01	2.02	2.01	0.01
4	MB4	Solid width 1 mm	1	1.02	0.98	0.98	≈0.99	−0.01
		Solid width 0.9 mm	0.9	0.92	0.93	0.91	0.92	0.02
		Solid width 0.8 mm	0.8	0.81	0.82	0.80	0.81	0.01
		Solid width 0.7 mm	0.7	0.72	0.71	0.68	≈0.70	0.00
		Solid width 0.6 mm	0.6	0.59	0.62	0.64	≈0.62	0.02
		Solid width 0.5 mm	0.5	0.50	0.52	0.54	0.52	0.02
		Solid width 0.4 mm	0.4	0.38	0.44	0.44	0.42	0.02
		Solid width 0.3 mm	0.3	0.34	0.36	0.33	≈0.34	0.04
		Solid width 0.2 mm	0.2	0.20	0.20	0.20	0.20	0.00
		Solid width 0.1 mm	0.1	0.10	0.10	0.13	0.11	0.01

**Table 7:** Comparison of selected parameters of the research prototypes.

Research Prototype		Selected Parameters		
		The Largest Absolute Difference between the Nominal Dimension and the Measured *	Average Values of Deviations between the Nominal Dimension and the Measured	The Smallest Absolute Difference between the Nominal Dimension and Measured *
Cylindrical	Cylinders Ø0.1 mm ÷ Ø10 mm	0.14 mm (Ø0.6; Ø0.4)	0.04 mm	0 mm (Ø4)
	Holes Ø0.1 mm ÷ Ø10 mm	0.10 mm (Ø0.1)	0.03 mm	0.01 mm (Ø10; Ø9; Ø5)
Rectangular	Rectangular holes width 0.1 mm ÷ 10 mm	0.10 mm (0.1)	0.05 mm	0 mm (3)
	Rectangular solids width 0.1 mm ÷ 10 mm	0.05 mm (8; 9)	0 mm	0 mm (6; 5; 4; 0.7; 0.2)

Comment: *—the parameters in parentheses refer to the feature that has reached the given dimension.

## Data Availability

The data presented in this study are available on request from the corresponding author.
